# Brief communication: Effects of conditioned media from human platelet lysate cultured MSC on osteogenic cell differentiation *in vitro*

**DOI:** 10.3389/fbioe.2022.969275

**Published:** 2022-09-29

**Authors:** Siddharth Shanbhag, Niyaz Al-Sharabi, Samih Mohamed-Ahmed, Reinhard Gruber, Einar K. Kristoffersen, Kamal Mustafa

**Affiliations:** ^1^ Department of Immunology and Transfusion Medicine, Haukeland University Hospital, Bergen, Norway; ^2^ Center for Translational Oral Research, Department of Clinical Dentistry, Faculty of Medicine, University of Bergen, Bergen, Norway; ^3^ Department of Oral Biology, University Clinic of Dentistry, Medical University of Vienna, Vienna, Austria; ^4^ Department of Periodontology, School of Dental Medicine, University of Bern, Bern, Switzerland; ^5^ Austrian Cluster for Tissue Regeneration, Vienna, Austria; ^6^ Department of Clinical Sciences, Faculty of Medicine, University of Bergen, Bergen, Norway

**Keywords:** mesenchymal stromal cells, conditioned media, platelet lysate, bone tissue engineering, osteogenic differentation

## Abstract

Culturing mesenchymal stromal cells (MSC) in human platelet lysate (HPL) supplemented media can enhance their osteogenic differentiation potential. The objective of this study was to test the hypothesis that conditioned media (CM) derived from HPL-cultured MSC also have pro-osteogenic effects. Pooled CM was prepared from HPL-cultured human bone marrow MSC (BMSC) of multiple donors and applied on BMSC of different donors (than those used for CM preparation), with or without additional supplementation [HPL, fetal bovine serum (FBS)] and osteogenic stimulation. At various time-points, cell proliferation, alkaline phosphatase (ALP) activity, osteogenic gene expression and *in vitro* mineralization were assessed. BMSC in standard unstimulated growth media served as controls. After 3–7 days, CM alone did not promote BMSC proliferation or ALP activity; supplementation of CM with HPL slightly improved these effects. After 2 and 7 days, CM alone, but not CM supplemented with HPL, promoted osteogenic gene expression. After 14 days, only CM supplemented with FBS and osteogenic stimulants supported *in vitro* BMSC mineralization; CM alone and CM supplemented with HPL did not support mineralization, regardless of osteogenic stimulation. In summary, CM from HPL-cultured BMSC promoted osteogenic gene expression but not *in vitro* mineralization in allogeneic BMSC even when supplemented with HPL and/or osteogenic stimulants. Future studies should investigate the role and relevance of supplementation and osteogenic induction in *in vitro* assays using CM from MSC.

## Introduction

Bone tissue engineering strategies are increasingly being used to overcome the limitations of autogenous bone grafts and existing biomaterials to reconstruct complex bone defects (Shanbhag et al., 2019). Conventional tissue engineering strategies involve the transplantation of autologous adult mesenchymal stromal cells (MSC)—usually from the bone marrow (BMSC), in combination with biomaterial scaffolds and/or signaling molecules at bone defect sites. However, certain limitations of this approach have been discussed. Firstly, in a recent meta-analysis, we found the clinical evidence for the effectiveness of this strategy to be limited; the effect sizes of cell therapy over traditional GBR or grafting procedures were relatively small and mainly limited to studies of maxillary sinus augmentation (Shanbhag et al., 2019). Secondly, large scale translation of autologous cell therapy is limited by the need for expensive Good Manufacturing Practice (GMP) grade laboratories for *ex vivo* cell expansion for each patient/production. Thirdly, the traditional hypothesis that MSC exert their bioactivity *via* engraftment, differentiation, and replacement at injury sites, has in recent years been challenged by evidence of a predominantly paracrine mechanism of action (Haumer et al., 2018).

It is now widely believed that MSC exert their effects *via* the secretion of a wide range of bioactive factors, including soluble proteins (growth factors, cytokines, chemokines), nucleic acids and microparticles [extracellular vesicles (EV)] at or near sites of injury (Gnecchi et al., 2016). These factors in turn stimulate tissue-resident progenitor (osteogenesis), endothelial (angiogenesis) and immune cells (immune modulation), to drive subsequent regeneration processes. Moreover, pre-conditioning or “priming” of MSC with various stimulants (growth factors, inflammatory cytokines, etc.) may further enhance their paracrine activity and immunomodulatory potential (Ferreira et al., 2018). These findings provide the biological basis for the development of “cell-free” strategies, which exploit the secretome contained in MSC conditioned media (CM) for tissue regeneration. A major advantage of this strategy is the possibility to produce secretomes on a large scale from a single (or limited) cell expansion cycle(s), and to use these factors as “off-the-shelf” products. The preclinical efficacy of MSC secretomes/CM for bone regeneration has recently been summarized (Veronesi et al., 2018; Benavides-Castellanos et al., 2020).

A critical aspect in the clinical translation of cell therapies is the use of safe and standardized culture conditions resulting in safe-to-use cell constructs. Exclusion of animal-derived supplements, e.g., fetal bovine serum (FBS), in *ex vivo* culture systems is considered important to facilitate clinical translation of cell therapies and is also a recommendation by regulatory health authorities (Bieback et al., 2019). This consideration may also be extended to cell-derivatives such as CM. Pooled human platelet lysate (HPL) has been identified as the optimal “xeno-free” supplement for MSC culture, with particular benefits for MSC osteogenic differentiation (Fekete et al., 2012; Shanbhag et al., 2017). We have recently reported that HPL-cultured MSC demonstrate superior proliferation, osteogenic gene expression and *in vitro* mineralization vs. corresponding FBS-cultured cells (Shanbhag et al., 2020a; Shanbhag et al., 2020b). Indeed, the type of supplement used to culture MSC can influence the composition and efficacy of their CM (Madrigal et al., 2014; Nikolits et al., 2021). In context, few studies have assessed the composition of CM from HPL-cultured MSC or compared the composition of CM from HPL- vs. FBS-cultured MSC (Kehl et al., 2019; Palombella et al., 2020; Kim et al., 2021). Several growth factors related to wound healing, angiogenesis and extra-cellular matrix production were found to be more abundant in the CM of HPL- vs. FBS-cultured BMSC (Kim et al., 2021). Thus, based on these data, it is reasonable to hypothesize that CM from HPL-cultured MSC may be more enriched and potentially pro-osteogenic.

In the context of bone tissue engineering, the efficacy of CM is often studied *in vitro via* its effects on MSC proliferation and osteogenic differentiation. In this regard, previous studies reported that CM promotes MSC osteogenic differentiation; CM in most cases, was derived from FBS-cultured MSC and applied on cells of rodent origin (see review Veronesi et al., 2018). However, for *in vitro* assays, CM is usually supplemented with serum since CM alone does not support longer term cell culture. For differentiation assays, usually lasting 14–21 days, CM is supplemented with both serum and osteogenesis-inducing supplements, i.e., L-ascorbic acid 2-phosphate, dexamethasone and/or β glycerophosphate, in various concentrations (Brauer et al., 2016). To our knowledge, no studies have tested the effects of CM from HPL-cultured human MSC on the osteogenic differentiation of human MSC, which would more closely simulate a clinical scenario. As previously discussed, it is reasonable to hypothesize that CM from HPL-cultured MSC may have pro-osteogenic effects. Thus, the main objective of this preliminary study was to investigate the effects of pooled CM derived from HPL-cultured human BMSC of multiple donors on the *in vitro* proliferation and osteogenic differentiation of allogeneic (different donor) BMSC. A secondary objective was to assess the need for additional supplementation and/or osteogenic stimulation in the *in vitro* assays.

## Methods

### Cell culture

The use of human cells and tissues was approved by the Regional Committees for Medical Research Ethics (REK) in Norway (2013-1248/REK-sør-øst and 2016-1266/REK-nord). Bone marrow specimens were obtained following parental consent from five independent donors (2 females and 3 males; 8–10 years) undergoing reconstructive surgery at the Department of Plastic Surgery, Haukeland University Hospital, Bergen, Norway; BMSC were isolated and expanded following previous protocols (Shanbhag et al., 2020b). Briefly, cells were cultured in T75 or T175 flasks (Thermo Fisher Scientific, Carlsbad, CA, United States) using sterile filtered growth media (GM) comprising of Dulbecco’s Modified Eagle’s medium (DMEM, Invitrogen, Carlsbad, CA, United States) supplemented with 5% (v/v) pooled human platelet lysate (HPL; Bergenlys, Bergen, Norway), 1% (v/v) penicillin/streptomycin (GE Healthcare, South Logan, UT, United States) and 1 IU/ml heparin (Leo Pharma AS, Lysaker, Norway). HPL was produced ‘in-house’ as described elsewhere (Shanbhag et al., 2020b). Cells were sub-cultured and expanded under standard incubation, i.e., 37°C and 5% CO_2_, according to a clinically validated protocol with a seeding density of 4000 cells/cm^2^ (Rojewski et al., 2019). Passage 1 (p1) and 2 (p2) BMSC were characterized based on immunophenotype and multi-lineage differentiation potential as previously reported (Shanbhag et al., 2020b), and used for CM preparation. In indicated experiments, BMSC from two separate donors (different from those used for CM preparation) were used to study the paracrine effects of CM. BMSC (p2) were seeded in 12-well plates (4000 cells/cm^2^) and exposed to CM for various durations in proliferation and differentiation assays. Cell attachment and morphology were regularly monitored under a light microscope (Nikon Eclipse TS100, Tokyo, Japan).

### Conditioned media preparation

CM was prepared from BMSC of three independent donors, as previously described (Al-Sharabi et al., 2017). Briefly, p1 and p2 BMSC were expanded in T175 flasks in GM until 70%–80% confluency under standard incubation. At this point, cells were washed three times with phosphate-buffered saline (PBS; Invitrogen) and then cultured in plain DMEM (without HPL or antibiotics) for another 48 h. After 48 h, CM from p1 and p2 BMSC from each of the three donors was collected, pooled, and centrifuged at 4000× *g* for 10 min to remove any debris. The supernatant was aliquoted and stored at −80°C. For all experiments, CM from −80°C storage was thawed overnight at 4°C and sterile filtered (0.2 μm) before use.

### DNA quantification and alkaline phosphatase activity assays

BMSC were seeded in 24-well plates at a density of 4000 cells/cm^2^ and cultured in GM. After 24 h, corresponding wells were washed with PBS and exposed to CM or CM-HPL (1% HPL); the concentration of HPL was adjusted in comparison to GM (5% HPL) to avoid overconfluency after 24 h. After 3 and 7 days, cells were lysed in 0.1% Triton X-100 (Sigma Aldrich) and DNA quantification and ALP activity assay were performed using the Quant-IT^®^ PicoGreen dsDNA Assay Kit (Thermo Fisher Scientific) and SIGMAFAST BCIP/NBT assay (Sigma-Aldrich), respectively, according to manufacturers’ instructions. DNA concentrations (ng/ml), calculated based on known standards, were used to normalize ALP activity of the corresponding cell-lysates.

### Gene expression analysis

Expressions of osteogenesis-related genes (Table 1) were assessed after 2 and 7 days *via* quantitative real-time polymerase chain reaction (qPCR) using TaqMan^®^ real-time PCR assays (Thermo Scientific). BMSC in GM were seeded in 12-well plates; after 24 h, corresponding wells were washed with PBS and exposed to different media formulations: GM, CM alone (CM) and CM supplemented with 5% HPL (CM-PL). RNA extraction and cDNA synthesis were performed as previously described (Mohamed-Ahmed et al., 2018) and expressions of genes of interest were normalized to that of a reference gene—glyceraldehyde 3-phosphate dehydrogenase (GAPDH). Data were analyzed by the ΔΔCt method and results are presented as fold changes relative to the reference group (GM) on a log(2)-transformed scale.

**TABLE 1:** Real time qPCR primers.

Gene (human)	TaqMan^®^ assay ID	Amplicon length
References		
GAPDH	Hs 02758991_g1	93
Osteogenesis-related		
RUNX2	Hs01047973_m1	86
COL1A2	Hs00164099_m1	68
OPN (SPP1)	Hs00959010_m1	84
OCN (BGLAP)	Hs01587814_g1	138

GAPDH glyceraldehyde 3-phosphate dehydrogenase, RUNX2 runt-related transcription factor 2, COL1A2 Collagen type 1 alpha 2, OPN/SPP1 osteopontin, OCN/BGLAP osteocalcin.

### *In vitro* mineralization assay

*In vitro* mineralization was assessed using the Alizarin red-S assay. BMSC in GM were seeded in 12-well plates; after 24 h, corresponding wells were washed with PBS and exposed to different media formulations with osteogenic induction supplements: growth media (GM+), CM (CM+) and CM with 5% HPL (CM-PL+). To induce osteogenic differentiation, media were supplemented with final concentrations of 0.05 mM L-ascorbic acid 2-phosphate, 10 nM dexamethasone and 10 mM β glycerophosphate (all from Sigma-Aldrich, St. Louis, MO, United States). Additionally, the following groups were included: CM supplemented with 2.5% HPL and osteogenic supplements and CM supplemented with 10% FBS and osteogenic supplements (CM-FBS+). After 14 days, formation of extracellular calcium deposits was assessed *via* Alizarin red S staining, as previously described (Mohamed-Ahmed et al., 2018). Briefly, after fixation with 4% paraformaldehyde, cells were stained with 2% Alizarin red S solution (Sigma Aldrich) for 30 min at RT, then washed and dried, before images were acquired.

### Statistical analysis

Statistical analysis was performed using the Prism 9 software (GraphPad Software, San Diego, CA, United States). Data are presented as means (± SD and/or range), unless specified. All linear data are presented as bar graphs. Normality testing was performed *via* the Shapiro-Wilk test. The student *t* test, Mann-Whitney U test, one-way analysis of variance (ANOVA; followed by a *post hoc* Tukey’s test) or Kruskal-Wallis test (followed by a *post hoc* Dunn’s test) were applied as appropriate, and *p* < 0.05 was considered as statistically significant.

## Results

### Conditioned media supplemented with platelet lysate did not enhance cell proliferation

**FIGURE 1:**
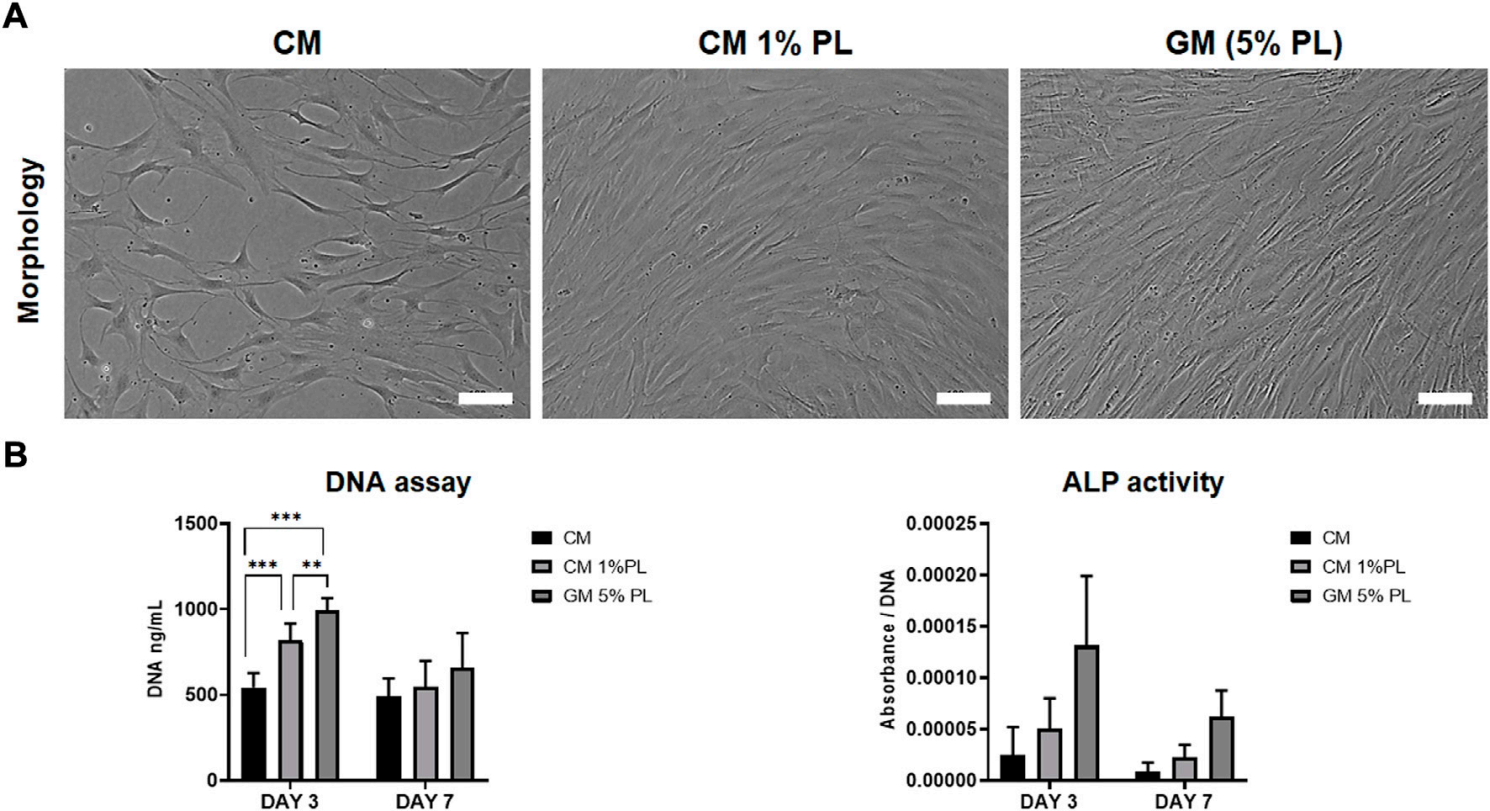
Proliferation and ALP activity. **(A)** Representative images of BMSC cultured in CM, CM supplemented with HPL (1% PL) or growth media (5% PL; control) after 3 days, scale bars 100 μm. **(B)** Quantification of total DNA (fluorescence) and ALP activity (absorbance) in BMSC cultured in CM, CM+1% PL and GM after 3 and 7 days (*n* = 2 donors; 3 experimental replicates per donor); **p < 0.005, ***p < 0.001.

The *in vitro* paracrine effects of CM were evaluated *via* proliferation and ALP activity assays using BMSC from two independent donors. DNA content of BMSC was lower in CM vs. GM (5% HPL) after 3 (*p* < 0.001) and 7 days (*p* > 0.05); supplementation of CM with 1% HPL did not attenuate this difference at 3 days (Figures 1A,B). A similar trend was observed for ALP activity between the groups, although without statistical significance (Figure 1B).

### Conditioned media supplemented with platelet lysate did not enhance osteogenic gene expression

**FIGURE 2:**
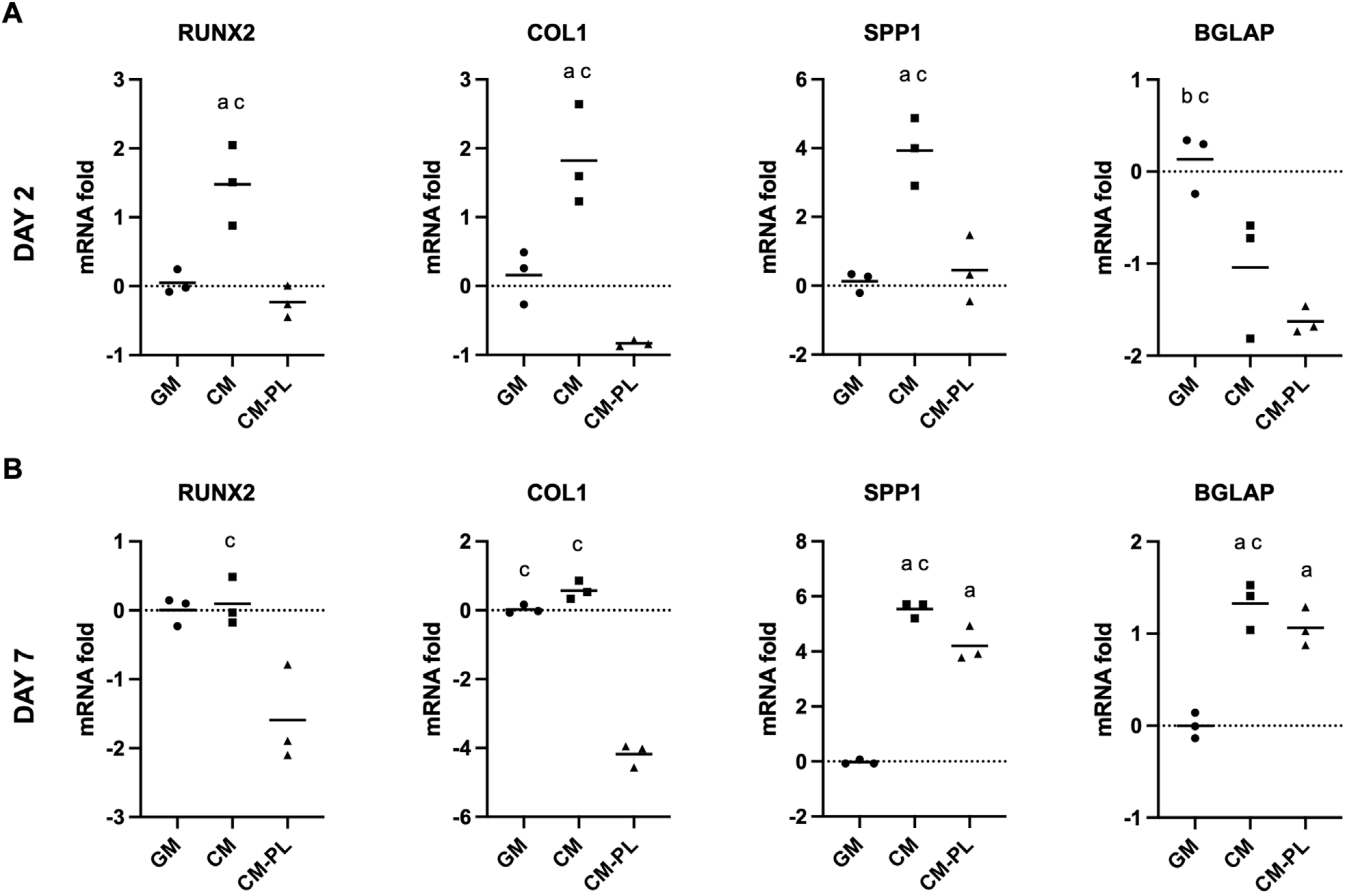
Osteogenic gene expression. Relative mRNA fold changes in BMSC after 2 **(A)** and 7 days **(B)**. Data indicate means of 3 experimental replicates for BMSC from one representative donor [Log(2)-transformed y-axis; negative values indicate gene downregulation]. Significance (*p* < 0.05) is denoted by alphabetical letterings: a, compared to GM; b, compared to CM; c, compared to CM-PL; GM, growth media; CM, conditioned media; conditioned media with platelet lysate.

After 2 d, compared to the reference group (GM), expressions of selected osteogenesis related genes, i.e., runt-related transcription factor 2 (RUNX2), collagen type 1A (COLIA), and osteopontin (SPP1/OPN), were significantly upregulated in BMSC exposed to CM alone after 2 and 7 days (*p* < 0.05 for all genes; Figures 2A,B). In particular, expression of SPP1/OPN was remarkably upregulated in CM. Expression of osteocalcin (BGLAP/OCN) was upregulated only after 7 days. When CM was supplemented with HPL (CM-PL), gene expression was either unchanged or downregulated compared to standard GM after 2 days. After 7 days, expressions of SPP1 and BGLAP were upregulated also in CM-PL (Figure 2B).

### Conditioned media supplemented with platelet lysate did not promote *in vitro* mineralization

**FIGURE 3:**
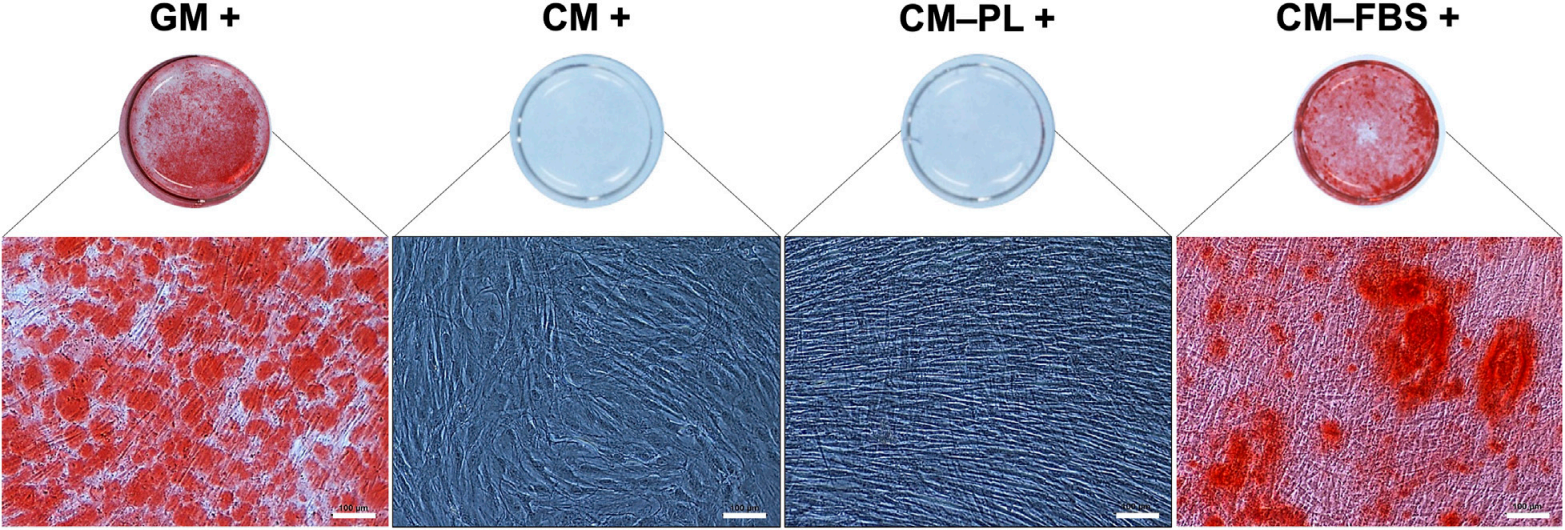
*In vitro* mineralization assay. Representative images of Alizarin red S stained BMSC after 14 days (*n* = 2 donors; 3 experimental replicates per donor), scale bars 100 μm. +, osteogenic induction added; GM, growth media; CM, conditioned media, PL, platelet lysate; FBS, fetal bovine serum.

Initially, for the *in vitro* mineralization assay, BMSC were exposed to CM alone or CM supplemented 5% HPL, both with osteogenic stimulants. After 14 days, no mineralization was observed in any of the test groups (Figure 3). Reduction of HPL concentration (from 5% to 2.5%) did not affect the results (data not shown). However, supplementation of CM with 10% FBS (CM-FBS+) revealed *in vitro* mineralization of BMSC comparable to the positive GM control (Figure 3).

## Discussion

Since HPL-cultured MSC demonstrate enhanced osteogenic differentiation and the CM of HPL-cultured MSC is more enriched than their FBS-cultured counterparts, the present study hypothesized that the CM of HPL-cultured BMSC has pro-osteogenic effects, i.e., the use of such CM could possibly reduce the need for additional serum/supplementation and/or osteogenic stimulation. Although previous studies have reported that CM promotes MSC osteogenic differentiation (Veronesi et al., 2018; Zhong et al., 2019), these have mainly assessed the effects of CM derived from FBS-cultured cells on MSC of non-human origin. Thus, little is known about the effects of CM from HPL-cultured MSC on allogeneic MSC of human origin. Such an *in vitro* setup would more closely simulate a clinical scenario of CM application, since: 1) current regulations recommend substitution of animal derivatives such as FBS in clinical-grade MSC cultures; and 2) CM would most likely be used as an allogeneic (pooled from multiple donors) “off-the-shelf” product. Therefore, the objective of this preliminary study was to investigate the effects of CM derived from HPL-cultured MSC (pooled CM from multiple donors) on the *in vitro* osteogenic differentiation of allogeneic (different donors) human MSC. The main findings were that: 1) CM alone promoted osteogenic gene expression, but not *in vitro* mineralization of BMSC, and 2) CM supplemented with HPL promoted neither osteogenic gene expression nor *in vitro* mineralization of BMSC.

In the present study, we investigated the influence of CM supplemented with different concentrations of HPL on the *in vitro* proliferation and osteogenic differentiation of BMSC. We observed that pure CM (without HPL) did not have positive effects on BMSC proliferation and *in vitro* mineralization, despite promoting significant upregulation of several osteogenesis-related genes. In previous studies, we showed that HPL-cultured MSC have an enhanced capacity for osteogenic differentiation (Shanbhag et al., 2020a; Shanbhag et al., 2020b). Therefore, we hypothesized that the secretomes of HPL-cultured BMSC (contained in CM) might have a stimulatory effect on cellular metabolic activity and osteogenic differentiation. However, based on the results herein, it seems that CM alone without added supplements (HPL/FBS) might impair the cultured BMSC *via* nutrient deprivation, thus reducing their metabolic activity and functions (Nuschke et al., 2016). It has also been reported that the “serum starvation” method used to collect CM might be associated with the lower content of the specific growth factors with metabolic activity in CM, e.g., hepatocyte growth factor (HGF), brain-derived neurotrophic factor (BDNF), and fibroblast growth factor 2 (FGF-2) (Petrenko et al., 2020). Further investigations to determine the effects of collection methods on the composition and concentrations of secreted molecules in CM are needed.

Cell metabolism and survival are controlled by the action of growth factors and cytokines through inhibition of apoptosis or promotion of cell survival. In previous studies, we have shown that CM from FBS-cultured BMSC contains several growth factors and cytokines with antiapoptotic and antioxidant properties, including platelet-derived growth factor (PDGF) and vascular endothelial growth factor A (VEGFA), and enhances *in vitro* osteogenic differentiation of MSC (Al-Sharabi et al., 2014; Al-Sharabi et al., 2016; Saleem et al., 2021). In context, HPL also contains several growth factors and cytokines, including PDGF, epidermal growth factor (EGF), insulin-like growth factor (IGF), transforming growth factor (TGF), FGF2, stem cell-growth factor-beta (SCGF), interleukin (IL)-1β, IL-2, -6, -10, -12p70, and IL-17A, tumor necrosis factor (TNF)-α and interferon (IFN) (Cañas-Arboleda et al., 2020; Shanbhag et al., 2020a). Therefore, it is reasonable to postulate that CM of HPL-cultured MSC would be correspondingly more enriched. Indeed, in a recent study, the concentrations of important growth factors such as VEGF, TGF-β1, and HGF were found to be significantly greater in the CM of HPL- vs. FBS-cultured MSC (Kim et al., 2021). However, we found that proliferation and osteogenic differentiation rates were insufficient in BMSC treated with 5% vs. 1% HPL-supplemented CM; a 5% concentration was selected based on current recommendations for HPL supplementation for *ex vivo* MSC expansion (Becherucci et al., 2018).

Regarding osteogenic differentiation, previous studies have reported that CM increased osteogenic differentiation and mineralization of MSC in a paracrine manner (Ogata et al., 2015; Zhong et al., 2019). However, most studies have not adequately addressed whether CM alone exerts this effect or whether the addition of FBS or HPL, with or without osteogenic supplements, is necessary. Such information would be important to standardize experimental setups and compare the results across different *in vitro* studies. In the present study, we found that pure CM stimulates neither ALP activity nor *in vitro* mineralization, as detected by Alizarin red staining. This was in line with a study conducted to evaluate proliferation and differentiation of osteoblasts under the induction of different concentrations of CM (Sun et al., 2012). When 1% HPL was added to CM, a slight improvement in ALP activity was detected although this was not equivalent to the control, i.e., GM containing 5% HPL. CM also promoted the expression of osteogenesis-related genes in BMSC, although this effect appears to be insufficient to stimulate *in vitro* mineralization, as no mineralization nodules were detected after 14 days. Thus, despite CM-induced gene upregulation in BMSC (without osteogenic supplementation), the impairment in promoting mineralization (with or without osteogenic supplementation) potentially reflects the safety of using BMSC as sources for CM production for different applications rather than specifically for bone regeneration.

In the context of osteogenic differentiation, we have previously reported that HPL supplementation alone (vs. FBS) enhances the expression of osteogenesis-related genes in MSC, suggesting particular benefits of HPL-supplemented MSC expansion for bone tissue engineering (Shanbhag et al., 2020a). Indeed, in the present study, exposure to CM resulted in an upregulation of osteogenic genes which was greater than that of HPL supplementation. However, the combination of HPL and CM did not exert a synergistic effect in terms of BMSC gene expression. This might indicate that a certain concentration of HPL together with CM might only allow the maintenance of the original microenvironment in BMSC, possibly *via* reduction of overexposure to cytokines and other stimulatory factors (Kandoi et al., 2018). On the contrary, the combination of CM and HPL may have antagonistic effects, which may distort the positive biological activity of CM. Therefore, future molecular research is warranted to study the effects of different combinational ratios of CM and HPL *in vitro* osteogenic differentiation of MSC (Aghamohamadi et al., 2020). Moreover, while the present study focused on osteogenic stimulatory capacity, other pathways of CM bioactivity, particularly angiogenesis (Quade et al., 2020) and immune-modulation (Jin et al., 2022), are also highly relevant for bone regeneration.

Some limitations of the present preliminary study must be acknowledged. The objective herein was to test the hypothesis that CM from HPL-cultured MSC may have pro-osteogenic effects, and not to compare per se CM from HPL- vs. FBS-cultured MSC or the osteogenic effects of CM supplemented with HPL vs. FBS. Therefore, we did not include FBS supplemented CM as a control group in all experiments, but only in the *in vitro* mineralization assay. Secondly, although the CM used herein was produced and pooled from multiple BMSC donors (*n* = 3), which is clinically relevant in terms of scaling up production and minimizing individual donor variations, the number of allogeneic BMSC donors for the *in vitro* assays was limited (*n* = 2). It is well known that BMSC may demonstrate significant donor-related variations in their growth and differentiation potential (Al-Sharabi et al., 2017) and therefore, the experiments should be repeated with BMSC from additional donors to validate the findings. Lastly, in this preliminary study, we did not perform any mechanistic assays, e.g., identification of specific signaling pathways, to determine the molecular basis for reduced mineralization in CM, with or without additional supplements. This would be relevant mainly for future *in vitro* assessments of MSC responses to CM to potentially predict (within the limitations of *in vitro* models) the *in vivo* effects of CM for bone regeneration. Clear descriptions of *in vitro* experimental setups, i.e., addition of serum/supplements and osteogenic stimulants, in future studies of CM efficacy are warranted to allow standardization and comparison of data.

## Conclusion

In summary, pooled CM from HPL-cultured human BMSC promoted osteogenic gene expression but not *in vitro* mineralization in allogeneic BMSC, even when supplemented with HPL and/or osteogenic stimulants. The role and relevance of CM supplementation and osteogenic stimulation in *in vitro* assays should be investigated in future studies to better understand the underlying molecular mechanisms and allow standardized comparisons of results.

## Data Availability

The original contributions presented in the study are included in the article/Supplementary Material, further inquiries can be directed to the corresponding author.
